# Absence of behavioural rhythms: Noise or unexplained neuronal mechanisms? (response to Fiebelkorn, 2021)

**DOI:** 10.1111/ejn.15628

**Published:** 2022-03-07

**Authors:** Sanne Ten Oever, Olof J. van der Werf, Teresa Schuhmann, Alexander T. Sack

**Affiliations:** ^1^ Language and Computation in Neural Systems Group Max Planck Institute for Psycholinguistics Nijmegen The Netherlands; ^2^ Donders Centre for Cognitive Neuroimaging Radboud University Nijmegen The Netherlands; ^3^ Department of Cognitive Neuroscience, Faculty of Psychology and Neuroscience Maastricht University Maastricht The Netherlands; ^4^ Maastricht Brain Imaging Centre (MBIC) Maastricht University Maastricht The Netherlands; ^5^ Department of Psychiatry and Neuropsychology, School for Mental Health and Neuroscience (MHeNs), Brain and Nerve Centre Maastricht University Medical Centre+ (MUMC+) Maastricht The Netherlands

**Keywords:** Egly–Driver task, replication, rhythmic attention, theta, visuospatial attention

Rhythmic neuronal patterns are omnipresent in the brain. It has been proposed that oscillations in the theta range govern visuospatial attention such that the brain sequentially samples different locations in space. If this were true, one would expect behavioural performance to follow the oscillatory sampling rhythm in the brain. Whereas some seminal papers have provided evidence for such behavioural sampling (Fiebelkorn et al., 2013; Landau & Fries, 2012), our recent paper did not show systematic rhythmic behavioural patterns at the cued location using similar design steps in one paper reporting this effect (Helfrich et al., 2018; Van der Werf et al., 2021). Given the previous evidence on the role of oscillations in attention sampling (Fiebelkorn et al., 2018; Szczepanski et al., 2014), we do not think that this absence of effect should be interpreted as an absence of the role of oscillations in attention, but rather as an eye‐opener to the sensitivity of these behavioural effects, as well as the still insufficient knowledge of how we can reliably study the role of oscillations in absence of electrophysiology.

Fiebelkorn (2021) argues that investigating the role of oscillations for behaviour critically depends on a high number of trials and the need of directly linking the behavioural data to ongoing oscillations for verifying the phase of stimulus presentation. It seems valid that the latter approach is successful. There is a plethora of studies showing that the phase of ongoing oscillations influence behaviour, not only in the domain of visuospatial attention (Fiebelkorn et al., 2018) but also for stimulus detection (Busch et al., 2009; Mathewson et al., 2009), auditory categorization (Hansen et al., 2019; Henry et al., 2016; Ten Oever & Sack, 2015) and memory (Batterink et al., 2016; Ten Oever et al., 2020). However, also in these other domains, purely behavioural oscillations independent of electrophysiology have been reported rather scarcely (de Graaf et al., 2013; Hickok et al., 2015; Jones, 1976; Ten Oever & Sack, 2015), with various null‐reports (Bosker & Kösem, 2017; Lin et al., 2020) and failed replications (Bauer et al., 2015). This begs to wonder whether all these studies simply lack a sufficient number of trials or whether they are rather indicative of a much more general issue, namely, that of approaching the problem in the wrong way. Ultimately, if we believe oscillations to be relevant and systematically related to behaviour, it seems valid to better understand why it is difficult to find these behavioural rhythms rather than to label the absence of an effect as (statistical) noise.

The difference between directly linking electrophysiological parameters to behaviour (linking studies) and behavioural oscillation studies (behavioural studies) is the way the noise enters the estimation of the oscillation parameters of interest (Figure 1). For linking studies, the noise of this estimation is solely dependent on the measurement noise of your electrophysiology and phase estimation ( $\varepsilon_{\text{link}}$). For example, volume conduction issues smear different sources into the estimation and the amount of electrical noise in the room or movement of your subject influences the estimation. Also, the way phase is extracted influences the reliability of the estimation. When estimating phase via the Hilbert transform, the phase estimation is dependent on the filter choices. For the fast Fourier transform, the phase estimation is dependent on choices in tapering and whether the signal is stationary or not. It is therefore not correct to state that electrophysiology always provides us with an estimation of the *true* phase of an ongoing oscillation. But we are indeed likely much closer to the actual oscillatory phase as compared with using a behavioural approach.

**FIGURE 1:**
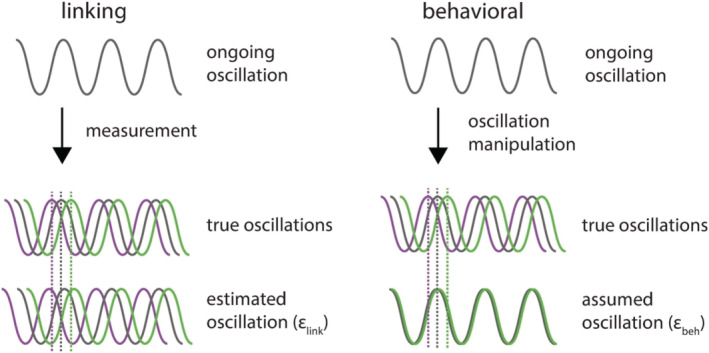
Noise ( ε) in the oscillatory estimation has different causes for linking studies and behavioural studies. Noise in linking studies is a consequence of an estimation error due to measurement noise and estimation errors. Noise in behavioural studies is a mismatch of the assumed oscillation and the true oscillation. Though $\varepsilon_{\mathrm{beh}}>\varepsilon_{\text{link}}$, we should try to minimize $\varepsilon_{\mathrm{beh}}$ as much as possible in order to improve sensitivity of behavioural oscillation studies

In a behavioural approach, it is necessary to have some form of oscillatory manipulation to modulate the neuronal oscillations in a predictable manner. In the sensory domain, this has been done by presenting either a high‐intensity phase reset event, such as a bright flash (Landau & Fries, 2012), or by presenting a sequence of rhythmic stimuli (Jones, 1976). One can also try to manipulate oscillations more directly using brain stimulation approaches such as TMS and tACS (de Graaf et al., 2020; Herrmann et al., 2013; Thut et al., 2011). One assumes that, through this oscillatory manipulation, one has systematically manipulated the phase of the oscillations (Figure 1). Therefore, presenting stimuli at different time points relative to this experimentally controlled oscillation should align to different oscillatory phases. Indeed, as Fiebelkorn (2021) pointed out, this assumption might be rather strong, and the variance of the phase might be much higher as compared with linking studies ( $\varepsilon_{\mathrm{beh}}>\varepsilon_{\text{link}}$).

We would argue, though, that we should strive for a better understanding of the origins of oscillations and how we can optimally manipulate them. As such, we can reduce the noise error due to the wrong assumption of the stability of the phase after the oscillatory manipulation. If we deem oscillations relevant, it is important to understand how we can externally manipulate them. Therefore, we can improve the sensitivity of these behavioural oscillation studies. Knowledge on how to manipulate oscillations also has a strong potential for usage in clinical interventions (Başar & Güntekin, 2008). An example of studying oscillatory manipulation is to investigate the best frequency to stimulate (Ali et al., 2013). The lack of understanding of how oscillatory manipulations work is evident from the various reported null results, and it is also apparent in our current study. For us, it remains an open question why the same phase reset manipulation is significant for non‐cued locations but not for cued locations (figure 4 in original paper), when the cue is moderately informative (80% cue validity). A better understanding of basic oscillatory dynamics is critical to answer these questions, which can in our view not only be attributed to insufficient statistical power or low number of trials.

In a similar vein, next to the noise when estimating oscillatory parameters, there is of course also noise in all studies that link other behavioural measures such as fatigue and sensitivity of the task to the assumed or extracted neuronal rhythms (Fielbelkorn, 2021). This in fact does not differ between linking and behavioural studies, but it is something to carefully consider when designing any experiment. Any distraction from the task will not pick up underlying attention fluctuations driven by the experimental manipulation. But there are also more subtle choices that matter. For example, whereas the most excitable phase of an oscillation might be most optimal for a detection task, a lower excitable phases might be more important for a discrimination task (Schaefer et al., 2006).

Ultimately, electrophysiology is the closest we can get to extracting oscillatory parameters and the gold standard in order to better understand oscillatory dynamics. To understand the relevance of these oscillations, however, it is unequivocal that we also have to link these dynamics to behaviour. We can use electrophysiological measures and directly link this to behaviour (Fiebelkorn, 2021), but we should also aim for more, that is, combining the knowledge of electrophysiology and computational modelling (Doelling & Assaneo, 2021; Roberts et al., 2013) for improving the designs for any study that aims to investigate behavioural rhythms in the absence of electrophysiology.

## CONFLICT OF INTEREST

No conflict of interest to declare.

### PEER REVIEW

The peer review history for this article is available at https://publons.com/publon/10.1111/ejn.15628.

## Data Availability

No new data or code were generated.
